# Stool Microbiota Diversity Analysis of *Blastocystis*-Positive and *Blastocystis*-Negative Individuals

**DOI:** 10.3390/microorganisms10020326

**Published:** 2022-01-31

**Authors:** Christen Rune Stensvold, Brede Aksdal Sørland, Rebecca P. K. D. Berg, Lee O’Brien Andersen, Mark van der Giezen, Joanna L. Bowtell, Ayman A. El-Badry, Salem Belkessa, Özgür Kurt, Henrik Vedel Nielsen

**Affiliations:** 1 Laboratory of Parasitology, Department of Bacteria, Parasites and Fungi, Statens Serum Institut, Artillerivej 5, DK-2300 Copenhagen S, Denmark; rebe@ssi.dk (R.P.K.D.B.); obi@ssi.dk (L.O.A.); hvn@ssi.dk (H.V.N.); 2 Department of Biology, Faculty of Science, University of Copenhagen, DK-2200 Copenhagen N, Denmark; fwp668@alumni.ku.dk; 3 Biosciences, University of Exeter, Exeter EX1 2LU, UK; mark.vandergiezen@uis.no; 4 Department of Chemistry, Bioscience and Environmental Engineering, University of Stavanger, 4021 Stavanger, Norway; 5 Department of Sport and Health Sciences, University of Exeter, Exeter EX1 2LU, UK; j.bowtell@exeter.ac.uk; 6 Department of Microbiology, College of Medicine, Imam Abdulrahman Bin Faisal University, Dammam 31451, Saudi Arabia; aelbadry@kasralainy.edu.eg; 7 Department of Biology, Faculty of Nature and Life Sciences, Ziane Achour University of Djelfa, Moudjbara Road, BP 3117, Djelfa 17000, Algeria; salembelkessa@yahoo.com; 8 Department of Medical Microbiology, School of Medicine, Acibadem Mehmet Ali Aydinlar University, Atasehir, Istanbul 34770, Turkey; oz1605@hotmail.com

**Keywords:** gut, parasite, *Blastocystis*, microbiome, microbiota, protist, ecology, eubiosis

## Abstract

*Blastocystis* is a unicellular eukaryote found in the gastrointestinal tract of both human and other animal hosts. The clinical significance of colonic *Blastocystis* colonization remains obscure. In this study, we used metabarcoding and bioinformatics analyses to identify differences in stool microbiota diversity between *Blastocystis*-positive and *Blastocystis*-negative individuals (n = 1285). Alpha diversity was significantly higher in *Blastocystis* carriers. At phylum level, Firmicutes and Bacteroidetes were enriched in carriers, while Proteobacteria were enriched in non-carriers. The genera *Prevotella*, *Faecalibacterium*, *Flavonifracter*, *Clostridium*, *Succinivibrio*, and *Oscillibacter* were enriched in carriers, whereas *Escherichia*, *Bacteroides*, *Klebsiella*, and *Pseudomonas* were enriched in non-carriers. No difference in beta diversity was observed. Individuals with *Blastocystis*-positive stools appear to have gut microbiomes associated with eubiosis unlike those with *Blastocystis*-negative stools, whose gut microbiomes are similar to those associated with dysbiosis. The role of *Blastocystis* as an indicator organism and potential modulator of the gut microbiota warrants further scrutiny.

## 1. Introduction

*Blastocystis* is an intestinal parasitic protist that has puzzled clinical microbiologists and gastroenterologists for decades. It is estimated that more than one billion people may be colonized by this organism [1]; in Denmark, we estimate that about 35% of the adult population are carriers [2]. Studies serving to unravel the role of *Blastocystis* in gastrointestinal health and disease have identified fecal microbiota diversity differences between individuals colonized by *Blastocystis* and those who are not [3,4,5,6,7]. However, the studies published so far have typically been limited by relatively small sample sizes, yielding conflicting results. In this study, the microbiota data from more than one thousand fecal samples from *Blastocystis*-positive (carriers) and *Blastocystis*-negative (non-carriers) individuals from various geographical regions were studied. We submitted all data obtained by metabarcoding to bioinformatics analysis to identify potential differences in fecal microbiota diversity. The results possibly suggest a role for *Blastocystis* as an indicator organism and a potential driver of gastrointestinal health and disease.

## 2. Materials and Methods

Sequence read data for a total of 1285 fecal DNAs generated by our in-house metabarcoding assay [8,9,10] for various independent research projects and routine diagnostic purposes were collated and analyzed using our in-house bioinformatics data pipeline previously described [8,9,11]. The fecal DNAs were from populations sampled in Algeria, Egypt, Turkey, United Kingdom, and Denmark. Data were fully anonymized; hence, no clinical or demographic data were available for study.

A sample was classified as positive for *Blastocystis*, if >1.5% of the total read count within the sample mapped to *Blastocystis* at genus level. We chose this positivity cut-off to avoid false positives resulting from sequencing contamination (carryover of ID tags) and because the assay has a preferential bias toward amplifying DNA from *Blastocystis* over other eukaryotic organisms. 

We carried out analyses for alpha (Shannon’s diversity index and richness) and beta (principal coordinate analysis (PCoA) of Bray–Curtis distances) diversity as well as linear discriminant analysis effect size (LEfSe) to identify and visualize taxonomic differences in microbiota signatures between carriers and non-carriers. Probability (*p*) values < 0.05 were considered to indicate statistically significant differences.

## 3. Results

Of the 1285 samples, for which data were available for this study, 235 (18.3%) were positive and 1050 (71.7%) were negative for *Blastocystis*. 

**Figure 1:**
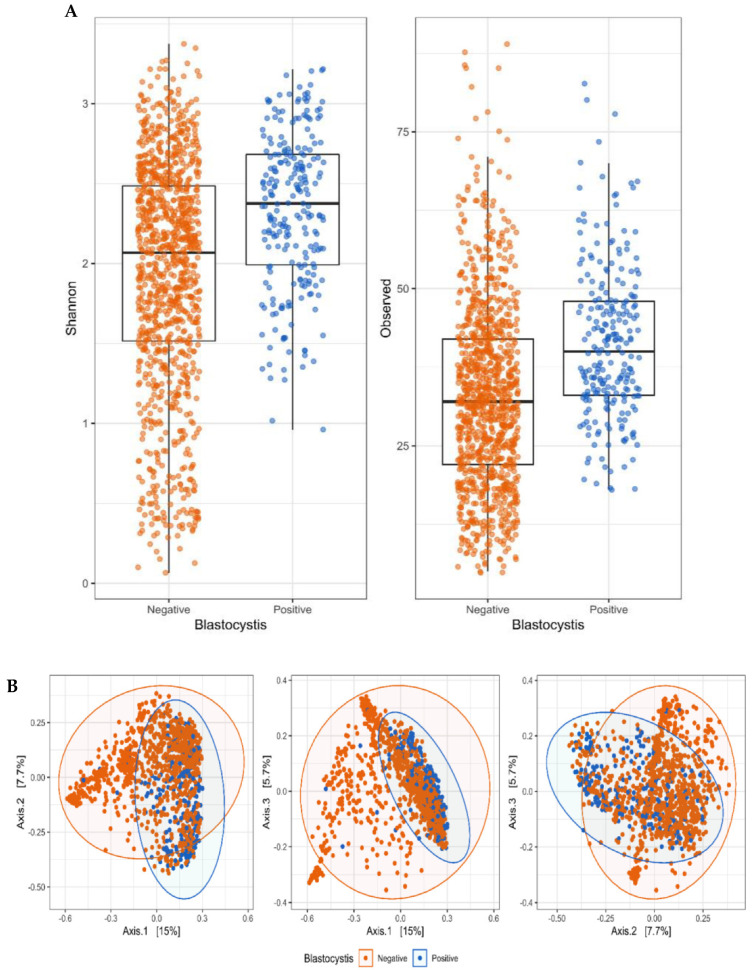
(**A**) Alpha diversity differences in Shannon’s diversity index (**left**) and observed richness (**right**) between *Blastocystis*-positive and *Blastocystis*-negative fecal DNA samples. Both measures differed significantly between the two types of samples (*p* < 0.0001). (**B**) Beta diversity of *Blastocystis*-positive and *Blastocystis*-negative fecal samples visualized by PCoA plots of Bray–Curtis dissimilarities.

Alpha diversity was significantly higher in the *Blastocystis*-positive samples than in the *Blastocystis*-negative samples for both Shannon diversity index (*p* < 0.0001) and observed richness (*p* < 0.0001) (Figure 1A).

With regard to beta diversity (Bray–Curtis; Figure 1B), no clear separation of the groups could be observed in the PCoA plot, suggesting no difference in beta diversity; however, there was a significant statistical difference between the two groups (*p* = 0.001). The statistically significant result may be explained by within-group variation, as the multivariate variation differed significantly (*p* < 0.0001) between groups. The fecal microbiota of carriers appeared to be more similar than that of non-carriers (Figure 1B).

**Figure 2:**
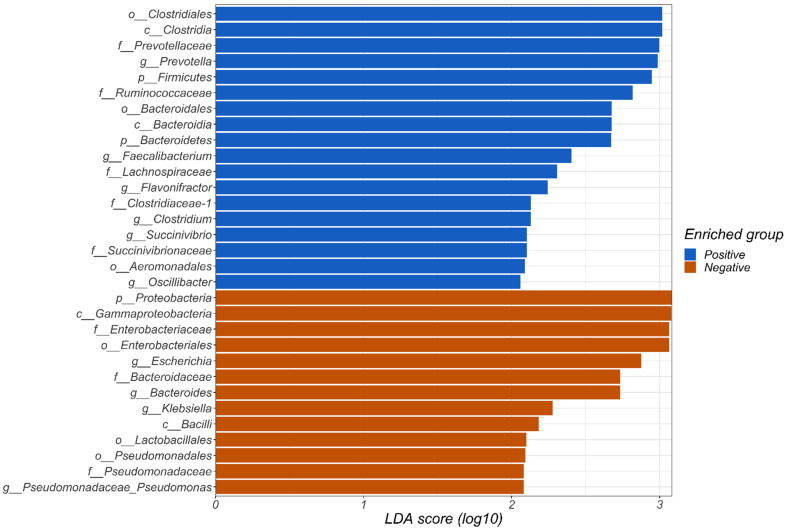
Linear discriminant analysis effect size (LEfSe) analysis of the microbiota in *Blastocystis*-positive and *Blastocystis*-negative fecal samples showing the bacterial taxa that were enriched in carriers and non-carriers, respectively. Taxa illustrated by blue bars are those enriched in *Blastocystis*-positive samples, whereas taxa illustrated by orange bars are those enriched in *Blastocystis*-negative samples. The small letters provided at the beginning of each taxon indicate the taxonomic tier (hence, ‘g’ is genus, ‘f’ is family, ‘c’ is class, etc.).

At phylum level, LEfSe analysis revealed that Firmicutes and Bacteroidetes were enriched in carriers, while Proteobacteria were enriched in non-carriers (Figure 2). At genus level, *Prevotella*, *Faecalibacterium*, *Flavonifracter*, *Clostridium*, *Succinivibrio*, and *Oscillibacter* among others were enriched in carriers, while *Escherichia*, *Bacteroides*, *Klebsiella*, and *Pseudomonas* were enriched in non-carriers, among others (Figure 2).

## 4. Discussion

Higher gut microbiome diversity appears to be conducive to gut health [12]. The higher bacterial diversity observed in individuals colonized by *Blastocystis* may indicate that these individuals typically have a healthier gut microbiota than those who do not carry the organism. In the healthy gut, metabolites such as short-chain fatty acids are developed by fermentation by anaerobic bacteria and possibly also by *Blastocystis* [13]. The metabolism of butyrate may favor a hypoxic intestinal lumen, which again favors colonization by *Blastocystis*, while disfavoring facultative anaerobic bacteria [13].

The enrichment in the families Prevotellaceae, Ruminococcaceae, and Clostridiaceae 1 in carriers supports our previous findings [3,4]. We have previously shown that *Blastocystis* is common in individuals with *Prevotella*- and *Ruminococcus*-driven enterotypes, whereas those with a *Bacteroides*-driven enterotype are rarely colonized [4]. 

In a metanalysis of 12 large metagenomics data sets, Beghini et al. [14] identified a generally higher abundance of the Bacteroidetes phylum in *Blastocystis*-negative samples, while identifying a strong enrichment of Firmicutes and Clostridiales in *Blastocystis* carriers.

In the present study, the Proteobacteria phylum was enriched in non-carriers as compared with carriers. Interestingly, another study suggested Proteobacteria as a potential diagnostic signature of dysbiosis and risk of disease [15]. Beghini et al. did not specifically report a consistent association between enrichment of Proteobacteria and absence of *Blastocystis*; however, based on the LEfSe analysis included in that study, both Gammaproteobacteria and Proteobacteria were 1000–10,000 times enriched in *Blastocystis*-negative samples [14], a finding that is in agreement with the observations of the present study (Figure 2). 

The present study was limited by the fact that no data on the health status of the tested individuals were available, which precluded investigation of the relationship between the presence of *Blastocystis* and specific factors associated with health and disease. Future studies should analyze microbiota data of carriers and non-carriers in the context of demographic and clinical data, such as functional and inflammatory bowel diseases. Nevertheless, given the findings presented here, those presented previously by our group [3,4,9], and those presented by independent research teams [5,6], it appears that *Blastocystis* could be considered a biomarker of intestinal eubiosis. Moreover, a recent study identified *Blastocystis* as an indicator of favorable postprandial glucose metabolism [16], and Beghini et al. recently identified a link between normal body mass index and *Blastocystis* colonization [14]. Further studies should couple bioinformatics analyses with clinical and demographic data and investigate whether *Blastocystis* is merely an indicator organism or also an active driver of gut microbiota diversity and host metabolism.

## Data Availability

Restrictions apply to the availability of these data. Data can be made available upon request.

## References

[1] Stensvold C.R., Tan K.S.W., Clark C.G. (2020). Blastocystis. Trends Parasitol..

[2] Krogsgaard L.R., Engsbro A.L., Stensvold C.R., Nielsen H.V., Bytzer P. (2015). The prevalence of intestinal parasites is not greater among individuals with irritable bowel syndrome: A population-based case-control study. Clin. Gastroenterol. Hepatol..

[3] Andersen L.O.B., Karim A.B., Roager H.M., Vigsnæs L.K., Krogfelt K.A., Licht T.R., Stensvold C.R. (2016). Associations between common intestinal parasites and bacteria in humans as revealed by qPCR. Eur. J. Clin. Microbiol. Infect. Dis..

[4] Andersen L.O.B., Bonde I., Nielsen H.B., Stensvold C.R. (2015). A retrospective metagenomics approach to studying Blastocystis. FEMS Microbiol. Ecol..

[5] Even G., Lokmer A., Rodrigues J., Audebert C., Viscogliosi E., Ségurel L., Chabé M. (2021). Changes in the human gut microbiota associated with colonization by *Blastocystis* sp. and *Entamoeba* spp. in non-industrialized populations. Front. Cell. Infect. Microbiol..

[6] Audebert C., Even G., Cian A., Loywick A., Merlin S., Viscogliosi E., Chabé M., El Safadi D., Certad G., Blastocystis Investigation Group (2016). Colonization with the enteric protozoa Blastocystis is associated with increased diversity of human gut bacterial microbiota. Sci. Rep..

[7] Nourrisson C., Scanzi J., Pereira B., NkoudMongo C., Wawrzyniak I., Cian A., Viscogliosi E., Livrelli V., Delbac F., Dapoigny M. (2014). Blastocystis is associated with decrease of fecal microbiota protective bacteria: Comparative analysis between patients with irritable bowel syndrome and control subjects. PLoS ONE.

[8] Stensvold C.R., Nielsen M., Baraka V., Lood R., Fuursted K., Nielsen H.V. (2021). Entamoeba gingivalis: Epidemiology, genetic diversity and association with oral microbiota signatures in North Eastern Tanzania. J. Oral Microbiol..

[9] Krogsgaard L.R., Andersen L.O.B., Johannesen T.B., Engsbro A.L., Stensvold C.R., Nielsen H.V., Bytzer P. (2018). Characteristics of the bacterial microbiome in association with common intestinal parasites in irritable bowel syndrome. Clin. Transl. Gastroenterol..

[10] Stensvold C.R., Lebbad M., Hansen A., Beser J., Belkessa S., Andersen L.O.B., Clark C.G. (2020). Differentiation of Blastocystis and parasitic archamoebids encountered in untreated wastewater samples by amplicon-based next-generation sequencing. Parasite Epidemiol. Control.

[11] Gotfred-Rasmussen H., Stensvold C.R., Ingham A.C., Johannesen T.B., Andersen L.O.B., Röser D., Nielsen H.V. (2021). Impact of metronidazole treatment and Dientamoeba Fragilis colonization on gut microbiota diversity. J. Pediatr. Gastroenterol. Nutr..

[12] Human Microbiome Project Consortium (2012). Structure, function and diversity of the healthy human microbiome. Nature.

[13] Stensvold C.R., van der Giezen M. (2018). Associations between gut microbiota and common luminal intestinal parasites. Trends Parasitol..

[14] Beghini F., Pasolli E., Truong T.D., Putignani L., Cacciò S.M., Segata N. (2017). Large-scale comparative metagenomics of Blastocystis, a common member of the human gut microbiome. ISME J..

[15] Shin N.R., Whon T.W., Bae J.W. (2015). Proteobacteria: Microbial signature of dysbiosis in gut microbiota. Trends Biotechnol..

[16] Asnicar F., Berry S.E., Valdes A.M., Nguyen L.H., Piccinno G., Drew D.A., Leeming E., Gibson R., Le Roy C., Khatib H.A. (2021). Microbiome connections with host metabolism and habitual diet from 1,098 deeply phenotyped individuals. Nat. Med..

